# Supplementary material for: NanoSafe III: A User Friendly Safety Management System for Nanomaterials in Laboratories and Small Facilities
Source: Nanomaterials (Basel). 2021 Oct 19;11(10):2768. doi: 10.3390/nano11102768 (PMC8541324; doi:10.3390/nano11102768)
Supplement: Supplementary file 1 [file nanomaterials-11-02768-s001.zip › nanomaterials-1406604-supplementary.pdf]

Supplementary Materials

NanoSafe III: A User Friendly Safety Management System for Nanomaterials in Laboratories and Small Facilities

Elina Buitrago [1,†], Anna Maria Novello [1,†], Alke Fink [2], Michael Riediker [3], Barbara Rothen-Rutishauser [2] and Thierry Meyer [4,*]

[1] Occupational Health and Safety (OHS), Station 6, Ecole Polytechnique Fédérale de Lausanne (EPFL), CH-1015 Lausanne, Switzerland; elina.buitrago@epfl.ch (E.B.); anna.novello@epfl.ch (A.M.N.)
[2] BioNanomaterials, Adolphe Merkle Institute, University of Fribourg, Ch. des Verdiers 4, 1700 Fribourg, Switzerland; alke.fink@unifr.ch (A.F.); barbara.rothen@unifr.ch (B.R.-R.)
[3] SCOEH: Swiss Centre for Occupational and Environmental Health, Binzhofstrasse 87, CH-8404 Winterthur, Switzerland; michael.riediker@alumni.ethz.ch
[4] Group of Chemical and Physical Safety (ISIC-GSCP), Station 6, Ecole Polytechnique Fédérale de Lausanne (EPFL), CH-1015 Lausanne, Switzerland
* Correspondence: Thierry.meyer@epfl.ch
† These authors contributed equally to the work.

Table S1. Hazard levels, threshold limit values and suggested occupational exposure limits for some common for ENM.

ENM	Hazard level	TLV Bulk (mg/m3) [SUVA]	Suggested OEL
Ni	H3	0.5	
Cu	H3	0.1	
Ag	H3	0.1<br>Ag metal dust: 0.01 mg/m³ (NIOSH)	0.190 μg/m³ [1]<br>0.33 μg/m³ [2,3]<br>0.67 μg/m³ [2,3]<br>0.098 μg/m³ [3]
Carbon black	H2	x	10 μg/m³ [4]<br>3.5 mg/m³ [5]<br>120 μg/m³ or 240 μg/m³ (different model) [6]<br>1 μg/m³ [7]
CNTs	H3	x	0.01 fibres/mL [8]<br>0.67 μg/m³ (MWCNT) [3]<br>1.2 μg/m³ (MWCNT) [2,9]<br>30 μg/m³ (CNT) [10]<br>50 μg/m³ (MWCNT Baytubes®) [11]
Fullerene	H1	x	7.4 μg/m³ [2]<br>390 μg/m³ [12]
SiO2 (amorphous)	H2	0.3	0.3 mg/m³ [13]
TiO2 (Anatase)	H1		0.3 mg/m³ [14]<br>0.017 mg/m³ [2]<br>0.1 mg/m³ [13]
TiO2 (Rutile)	H2	3<br>0.6 mg/m³ (Japan)	0.61 mg/m³ [15]<br>73ug/m³ [6]<br>1mg/m³ (high surface reactivity anatase-rutile nanoscale) [16]<br>2mg/m³ (low surface reactivity nanoscale TiO_2 [16]
Iron oxide (Fe_3O_4)	H1	3	iron oxide (Fe_3O_4)

ZnO	H2	3	20000particles/cm3 [insoluble] [8]
MnO	H2		0.013 mg/m^3 [insoluble] [8]
CoO	H2		0.033 mg/m^3 [insoluble] [8]
Ag2O	H2		0.0066 mg/m^3 [insoluble] [8]
Ag2O	H2		0.005 mg/m^3 [soluble] [8]
Al2O3	H2		20000particles/cm^3 [insoluble] [8]

Ostwald–Freundlich equation for calculating solubility of a nanomaterial smaller than 10 nm:

$$S = S_0 \frac{2\gamma V}{RTr} \quad (1)$$

S is the solubility (in mol kg-1) of spherical particles
R is the radius (m)
S_0 is the solubility of the bulk
V is the molecular volume (m^3 mol^{-1})
γ is the surface tension (J m^{-2}). The typical value for oxides is 0.5 J
R is the gas constant 8.314 (J/mol K)
T is the temperature (K)

References

1. Weldon, B.A.; M. Faustman, E.; Oberdörster, G.; Workman, T.; Griffith, W.C.; Kneuer, C.; Yu, I.J. Occupational Exposure Limit for Silver Nanoparticles: Considerations on the Derivation of a General Health-Based Value. *Nanotoxicology* **2016**, *10*, 945–956, doi:10.3109/17435390.2016.1148793.
2. Aschberger, K.; Micheletti, C.; Sokull-Klüttgen, B.; Christensen, F.M. Analysis of Currently Available Data for Characterising the Risk of Engineered Nanomaterials to the Environment and Human Health — Lessons Learned from Four Case Studies. *Environment International* **2011**, *37*, 1143–1156, doi:10.1016/j.envint.2011.02.005.
3. Stone, V.; Hankin, S.; Aitken, R.; Aschberger, K.; Baun, A.; Christensen, F.; Fernandes, T.; Hansen, S.F.; Hartmann, N.I.B.; Hutchinson, G. *Engineered Nanoparticles: Review of Health and Environmental Safety (ENRHES). Project Final Report*; European Commission, 2010;
4. Świdwińska-Gajewska, A.M.; Czerczak, S. Nanosilver – Occupational exposure limits. *Med Pr* **2015**, *66*, 429–442, doi:10.13075/mp.5893.00177.
5. CDC - NIOSH Pocket Guide to Chemical Hazards - Carbon Black Available online: https://www.cdc.gov/niosh/npg/npgd0102.html (accessed on 14 September 2021).
6. Kuempel, E.D.; Tran, C.L.; Castranova, V.; Bailer, A.J. Lung Dosimetry and Risk Assessment of Nanoparticles: Evaluating and Extending Current Models in Rats and Humans. *Inhalation Toxicology* **2006**, *18*, 717–724, doi:10.1080/08958370600747887.
7. NIOSH *Current Intelligence Bulletin 65: Occupational Exposure to Carbon Nanotubes and Nanofibers*; NIOSH, 2013;
8. Benke, G.; Dennekamp, M.; Priestly, B.; Sim, M. Engineered Nanomaterials: Feasibility of Establishing Exposure Standards and Using Control Banding in Australia. **2010**.
9. Aschberger, K.; Johnston, H.J.; Stone, V.; Aitken, R.J.; Hankin, S.M.; Peters, S.A.K.; Tran, C.L.; Christensen, F.M. Review of Carbon Nanotubes Toxicity and Exposure—Appraisal of Human Health Risk Assessment Based on Open Literature. *Critical Reviews in Toxicology* **2010**, *40*, 759–790, doi:10.3109/10408444.2010.506638.
10. Nakanishi, J. *Risk Assessment of Manufactured Nanomaterials*; 2011;
11. Pauluhn, J. Subchronic 13-Week Inhalation Exposure of Rats to Multiwalled Carbon Nanotubes: Toxic Effects Are Determined by Density of Agglomerate Structures, Not Fibrillar Structures. *Toxicological Sciences* **2010**, *113*, 226–242, doi:10.1093/toxsci/kfp247.
12. Morimoto, Y.; Kobayashi, N.; Shinohara, N.; Myojo, T.; Tanaka, I.; Nakanishi, J. Hazard Assessments of Manufactured Nanomaterials. *Journal of occupational health* **2010**, *52*, 325–334.
13. Stockmann-Juvala, H.; Taxell, P.; Santonen, T. *Formulating Occupational Exposure Limit Values (OELs) (Inhalation & Dermal)*; p. 20;.
14. NIOSH *Current Intelligence Bulletin 63: Occupational Exposure to Titanium Dioxide*; NIOSH, 2011;
15. Hanai, S.; Kobayashi, N.; Ema, M.; Ogura, I.; Gamo, M.; Nakanishi, J. Risk Assessment of Manufactured Nanomaterials–Titanium Dioxide (TiO2). *NEDO project "Research and development of nanoparticle characterization methods"(P06041)* **2009**.
16. Warheit, D.B. How to Measure Hazards/Risks Following Exposures to Nanoscale or Pigment-Grade Titanium Dioxide Particles. *Toxicology Letters* **2013**, *220*, 193–204, doi:10.1016/j.toxlet.2013.04.002.
